# Hybrid Plasmonic Symmetry‐Protected Bound state in the Continuum Entering the Zeptomolar Biodetection Range

**DOI:** 10.1002/smll.202411827

**Published:** 2025-01-26

**Authors:** Elena Clabassi, Gianluca Balestra, Giulia Siciliano, Laura Polimeno, Iolena Tarantini, Elisabetta Primiceri, David Maria Tobaldi, Massimo Cuscunà, Fabio Quaranta, Adriana Passaseo, Alberto Rainer, Silvia Romano, Gianluigi Zito, Giuseppe Gigli, Vittorianna Tasco, Marco Esposito

**Affiliations:** ^1^ CNR NANOTEC Institute of Nanotechnology Via Monteroni 73100 Lecce Italy; ^2^ Department of experimental medicine University of Salento Lecce 73100 Italy; ^3^ CNR IMM Institute for Microelectronics and Microsystems Via Monteroni 73100 Lecce Italy; ^4^ CNR ISASI Institute of Applied Sciences and Intelligent Systems Naples 80078 Italy; ^5^ Department of Engineering University Campus Bio‐Medico di Roma via Álvaro del Portillo 21 Rome 00128 Italy

**Keywords:** bound states in the continuum, hybrid metasurfaces, hybrid SP‐BICs, localized surface plasmon resonances, zeptomolar biosensing

## Abstract

Photonics bound states in the continuum (BICs) are peculiar localized states in the continuum of free‐space waves, unaffected by far‐field radiation loss. Although plasmonic nano‐antennas squeeze the optical field to nanoscale volumes, engineering the emergence of quasi‐BICs with plasmonic hotspots remains challenging. Here, the origin of symmetry‐protected (SP) quasi‐BICs in a 2D system of silver‐filled dimers, quasi‐embedded in a high‐index dielectric waveguide, is investigated through the strong coupling between photonic and plasmonic modes. By tailoring the hybridizing plasmonic/photonic fractions, a trade‐off is selected at which the quasi‐BIC exhibits both high intrinsic Q‐factor and strong near‐field enhancement because of dimer‐gap hotspot activation. Not only radiation loss is damped but in a configuration sustaining a lattice of plasmonic hotspots. This leads to an advantageous small modal volume for enhancing light‐matter interaction. The layout of nearly embedded dimers is designed to maximize the spatial overlap between the optical field and the target molecules, enhancing reactive sensing efficiency. The architecture is evaluated for its ability to detect transactive response DNA‐binding protein 43. The refractometric sensitivity outperforms current label‐free biosensing platforms, reaching the zeptomolar range. The approach highlights the potential of combining plasmonic and dielectric nanomaterials for advanced sensing technologies.

## Graphical abstract

The origin of hybrid symmetry‐protected quasi‐bound states in the continuum (SP‐BIC) is explored by envisaging a new functional optical platform combining photonic/plasmonic modes in a non‐trivial 2D nanoarchitecture. The engineered building block sets the optical conditions to enter in the strong coupling regime, unlocking hybrid‐BIC states with high Quality factor and small modal volume (V) driven by nanoscale hotspot activation, demonstrating exceptional biosensing performance toward zeptomolar‐level sensitivity.

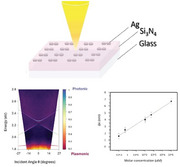

## 1 Introduction

Photonics bound states in the continuum (BICs) have attracted significant attention due to their remarkable properties. BICs have been proved to be a novel method of light confinement, coexisting within the continuous spectrum of radiating waves, but remaining inaccessible to the far field owing to their topological mechanism of protection from radiation loss. This is why BICs are additionally referred to as embedded or trapped modes and characterized by theoretical infinite quality factors (Q).^[^ ^1^, ^2^ ^]^ Symmetry‐protected (SP) BICs have received great attention. They emerge at high symmetry points, like the Γ‐point in the reciprocal space of a periodic structure, and cannot couple with free‐space radiative waves because of mode symmetry mismatch. The topological nature of these modes, arising from their polarization singularity^[^ ^3^ ^]^ makes them robust against defects or structure perturbations. Their high radiative quality factor (*Q*_r_) has made them a promising tool in a variety of applications such as lasing,^[^ ^4^, ^5^ ^]^ filtering,^[^ ^6^ ^]^ non‐linear device^[^ ^7^ ^]^ and sensing.^[^ ^8^ ^]^ In real structures, quasi BICs have large but finite radiative losses.^[^ ^9^ ^]^ In particular, breaking in‐plane inversion symmetry allows engineering radiation loss to match nonradiative material loss to achieve the critical coupling condition at which the local field enhancement is maximized.^[^ ^10^, ^11^ ^]^ However, this strategy can lead to highly reduced radiative intrinsic *Q* factor, such as 150 even in pure dielectric materials like silicon^[^ ^12^ ^]^ (where $Q^{-1}={Q_{\mathrm{nr}}}^{-1}+Qr^{-1}$). Since light‐matter interaction is ruled by the ratio *Q*/*V*,^[^ ^13^ ^]^ where *V* measures the effective mode volume, squeezing the electromagnetic field of a quasi‐BIC into plasmonic hot spots in a cooperative fashion could be advantageous.^[^ ^14^, ^15^, ^16^ ^]^

The hybridization of electromagnetic modes of different natures offers a promising approach.^[^ ^17^, ^18^ ^]^ Pure plasmonic modes can confine light at subwavelength scales, but their resonances typically suffer from low *Q*‐factor as damped by poor nonradiative Q_nr_. In this regard, the radiation engineering approach offered by BICs is particularly beneficial for plasmonic nanoparticles, which not only experience significant ohmic losses–affecting Q_nr_ especially in the visible range—but also suffer from high radiative losses and hence reduced Q_r_. By enhancing their radiative quality factor, plasmonic quasi BICs represent a fertile platform for improved light‐matter interaction that has enabled considerable applications.^[^ ^19^, ^20^, ^21^ ^]^ Notably, quasi BIC nanostructures leveraging innovative structural designs aimed at high‐sensitivity solutions are gaining prominence in the biosensing field,^[^ ^22^, ^23^ ^]^ achieving remarkably low limits of detection.^[^ ^24^, ^25^, ^26^, ^27^, ^28^, ^29^ ^]^ Therefore, the hybridization of plasmonic and photonic modes has been shown to effectively address several limitations.^[^ ^17^, ^18^, ^30^ ^]^ Additionally, off‐**Γ** point hybrid photonic–plasmonic BICs, following Friedrich‐Wintgen condition, have been explored, as demonstrated in recently reported 1D and 2D systems.^[^ ^19^, ^20^, ^31^, ^32^, ^33^, ^34^, ^35^, ^36^ ^]^

Herein, we engineer a metallo‐dielectric metasurface capable of sustaining a hybrid quasi‐bound state in the continuum arising from the strong coupling of the plasmonic and photonic modes, thus resulting in a significantly enhanced *Q*‐factor of 250 compared to conventional plasmonic nanostructures and even pure plasmonic quasi‐BICs in the mid‐IR range.^[^ ^37^ ^]^ Our system consists of silver‐nanoparticle dimers embedded in a silicon nitride (Si₃N₄) waveguide. This design not only supports high‐*Q* resonances but also a plasmonic hot spot with a small modal volume (*V)*, which synergically maximizes the electromagnetic field at the plasmonic/dielectric interface. This strategic field localization enhances interaction with target molecules, overcoming the limitations of purely dielectric structures, where the field is confined within the inner volume. Instead of breaking in‐plane inversion symmetry for far field coupling and critical radiative‐nonradiative matching conditions, we leverage the incorporated dimer antennas for efficiently injecting energy into the system through their strong dipolar radiative coupling. We demonstrate the emergence of this quasi BIC in the visible range, advantageous for technical applications. The BIC is polarization‐driven and influenced by the detuning of energy (DE) between the guided mode and the localized surface plasmon resonance (LSPR) of the silver dimer. Our architecture exhibits multifunctional properties enabled by its intrinsic configurational features, enabling the desired high *Q/V* ratio and strong local field enhancement in the plasmonic gap hot spot. We show that operating in a DE region that optimizes the balance between high *Q* and low *V* allows to achieve large sensitivity. This optimization is achieved through fine‐tuning of the plasmonic and photonic fractions of the coupled resonances by varying the lattice period (LP). Moreover, the 2D features of the system grant direct access to the active surface for light‐matter applications, in particular biosensing. Indeed, the system, in combination with a bio‐inspired reactive coating, was exploited for the detection of a biomarker associated with neurodegenerative diseases, the transactive response (TAR) DNA‐binding protein 43 (TDP‐43), allowing its optical detection up to zeptomolar concentrations. To the best of our knowledge, such a high sensitivity represents the first biosensing signature reaching this detection level by exploiting BIC technology.

## 2 Results

**Figure 1:**
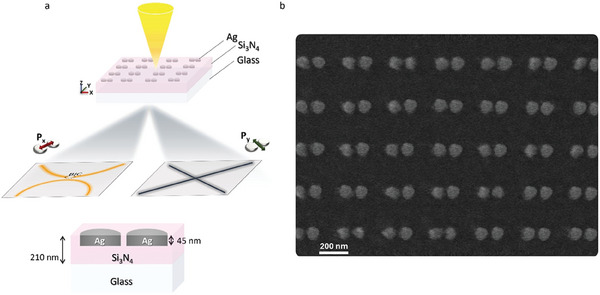
a) Schematic view of the dimer‐based nanoarchitecture supporting hybrid BIC generation in a switchable configuration depending on incident light polarization. b) SEM image (top view) of the fabricated dimer array embedded in the Si3N4 waveguide. The square arrays of silver nanoparticle dimers (70 nm ND diameter, 320 nm lattice period along the x‐axis, 300 nm lattice period along the y‐axis, 45 nm thickness, and 30 nm gap) have been realized inside a Si3N4 slab of 210 nm thickness by a combination of PECVD, electron beam lithography, ICP RIE, and thermal evaporation, as detailed in the experimental section.

To demonstrate the optical emergence of a plasmonic symmetry‐protected quasi‐BIC we numerically and experimentally developed a new paradigm of a quasi‐embedded metasurface consisting of a square array of fully silver‐filled nanoholes dimers inside a Si_3_N_4_ waveguide slab (**Figure** **1**) (see Experimental Section). This choice can be particularly effective in view of biosensing applications since the dimers design allows to achieve enhanced plasmonic field (within the nanogap), while the surface air exposure enables easy access to the plasmonic field and maximizes its superposition with biomaterials. In this matter, it is noteworthy that present state‐of‐the‐art designs are limited to plasmonic components either covered entirely by dielectric claddings^[^ ^19^, ^32^ ^]^ or placed on top of a waveguide layer.^[^ ^33^, ^34^ ^]^

### 2.1 Design of Dimer‐Based Architecture for Hybrid SP‐BIC Generation

A non‐radiative BIC shows up at the gamma point of a 2D dielectric array when it has sub diffractive lattices and the nanoparticles support only the in‐phase resonant out‐of‐plane dipole modes.^[^ ^38^ ^]^ These oscillating dipoles along the normal incident direction do not radiate toward the far field, whereas destructive interference in all other directions limits external radiative loss.^[^ ^39^ ^]^

It is possible to create a radiation channel that transforms the BIC mode from a purely sub diffractive state into a leaky resonance (quasi‐BIC) with a high finite radiative Q‐factor. This can be achieved by precisely adjusting one of the periods to support a diffraction order at the wavelength of the nanoobject's resonance, or by establishing a refractive index contrast around the nanoparticles, such as embedding them within a high‐index dielectric waveguide.^[^ ^35^ ^]^

In our case, following the latter approach (dielectric waveguide in silicon nitride), when incident light hits the subwavelength structure, it couples to guided modes following their dispersion curves in the energy‐momentum space. We numerically investigate the effects of the 2D lattice on the slab‐guided modes. The coupling between these modes and the first diffractive order leads to the avoided crossing dispersion bands and BIC emergence for both incident polarizations (Figure S1, Supporting Information).^[^ ^3^, ^40^ ^]^

Moving away from the BIC position, the non‐zero radiative losses take place scaling with momentum displacement.^[^ ^10^ ^]^ To date, these optical quasi states have been used for various applications. This is because the high Q value balances the large mode volume V (low field enhancement (M)) of the all‐dielectric nanostructure resonance in the Q/V ratio. This ratio defines the metrics for nanoscale enhancement.^[^ ^41^ ^]^ Anyway, to reach high Purcell factor values, the electromagnetic field has to be strongly enhanced. This motivates the research for hybrid guided plasmonic modes where the optical near field can be strongly enhanced by a subwavelength plasmonic component while still preserving low‐losses high Q factor characteristics of the dielectric counterpart.^[^ ^33^ ^]^

For this purpose, we filled the dimers‐arranged nanoholes with silver. The metal's choice is driven by the possibility of observing its optical response in the visible range (Figure S2, Supporting Information). The LSP resonances sustained by the metallic nanostructures enable the coupling with the BIC‐like photonic branches leading to modes splitting and the formation of a hybrid photonic/plasmonic quasi BIC mode. This design demonstrates remarkable adaptability, as structural parameters such as lattice period and nanogap size (Figure S3, Supporting Information) can be finely tuned, along with the orientation of incident light polarization relative to the dimer axis. In sharp contrast, silver dimers merely positioned atop the waveguide fail to achieve the coupling regime required to realize strong‐coupled quasi‐BICs, as illustrated in Figure S4 (Supporting Information). This underscores the critical importance of overcoming the technological challenge of embedding silver nanoparticles within the dielectric waveguide. Our approach with nearly embedded dimers is then pivotal for maintaining an accessible surface for molecular adsorption, thus combining advanced optical properties with practical application potential.

**Figure 2:**
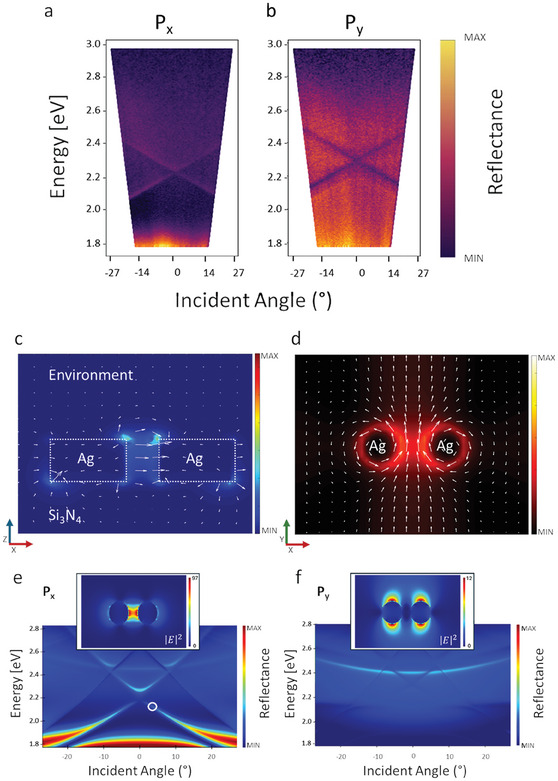
a,b) Measured angular reflectance maps as a function of the incident angle (θ°) for incident light polarization parallel (P_x_) and normal (P_y_) to the dimer axis. c,d) Electric and magnetic field vectors (arrows) and normalized intensities calculated at the quasi‐BIC point for polarization P_x_. e,f) Numerical simulation of the reflectance maps. In the insets, the near‐field distributions of the electric field are shown, corresponding to the quasi‐BIC point circled in the map. The value of | ***E*** |^2^ **shown is normalized to the incident electric field |E_0_|.^2^

In order to gain insight into the features of the hybrid BICs, in **Figure** **2**a,b, we plot the measured reflectance spectra as a function of the incident angle (θ^○^) for incident light polarization parallel and normal to the dimer axis, respectively indicated as *P_x_* and *P*_y_. Along the *P*_x_ polarization, we noted the emergence of the symmetry‐protected hybrid BIC at 2.2 eV triggered by the dimer LSPR signature that is redshifted with respect to the photonic modes illustrated in Figure S2a (Supporting Information). In this case, although the DE between the modes is non‐zero, the spatial overlap and intensity of the electric fields involved, namely the plasmonic field in the nanogap and the photonic BIC, lead to strong coupling and hybrid BIC generation.

The physical origin of the BIC state is further identified by the corresponding electric and magnetic field distributions calculated at the quasi‐BIC point, displayed in Figure 2c,d. The electric field vectors within the *xz*‐plane point outward from the dimers and normally to silver atop the surface. The magnetic field vectors revolve around the silver islands, in agreement with other reports.^[^ ^20^ ^]^ Switching to the *P*_y_ polarization (Figure 2b), the LSPR is centered at 2.3 eV (Figure S2b, Supporting Information) and emerges from the single nanoparticle dimer, spectrally overlapping with the photonic modes. Here, the reflectance map shows only dark crossing modes indicating that no BIC onset is triggered, owing to a reduced electric field strength of the single nanoparticle dimer.

The electric field distributions calculated at the two considered polarizations are displayed in the insets of Figure 2e,f and allow us to highlight the effect of the dimer gap as we move from the P_x_ case, where the field is more confined inside the gap, to the Py polarization where it is highly delocalized and around the dipolar lobes of the two disks. Also, the intensity value of the field is remarkably enhanced along P_x_ with respect to the opposite polarization, sustaining the claim that the latter is too weak to trigger the hybrid BIC generation.

Following this analysis, we can argue that in a strong coupling regime characterized by limited detuning energy the high LSP losses act as an additional destructive leaky channel overcoming the Rabi Splitting $\Omega <\frac{\left(\gamma_{LSP}-\gamma_{GM}\right)}{2}$, where γ_*LSP*_ and γ_*GM*_ represent the LSP and guided modes losses, respectively, This issue is further exacerbated by the lack of spatial overlap between the plasmonic and photonic electric fields, preventing effective coupling. Without such overlap, the electromagnetic modes remain isolated, inhibiting the formation of a coherent hybrid state necessary for achieving strong coupling.

Figure S5 (Supporting Information) shows further details of the quasi‐BIC, in particular the eigenpolarization map calculated propagating in the far field, showing a topological charge of 1 given by the rotation of the azimuthal angle of the polarization ellipse with a characteristic V‐point polarization singularity.^[^ ^3^ ^]^ Finally, Figure S6 (Supporting Information) shows the extracted Fano profile of the hybrid quasi‐BIC.^[^ ^42^ ^]^

To clarify the optical behavior of our system in the hybrid BIC generation along the dimers axis, we described the obtained dispersion and anti‐crossing modes by a three‐coupled harmonic oscillators model according to the following Hamiltonian:

(1) $$H\;\left(\begin{array}{ccc} E_{ph}^{+} & 0 & \frac{\Omega }{2}\\ 0 & E_{ph}^{-} & \frac{\Omega }{2}\\ \frac{\Omega }{2} & \frac{\Omega }{2} & E_{LSPR} \end{array}\right)$$

where *E_LSPR_* is the plasmon energy, Ω is the Rabi splitting and $E_{ph}^{\pm }$ are the energy dispersions of the BIC‐like photonic branches.

**Figure 3:**
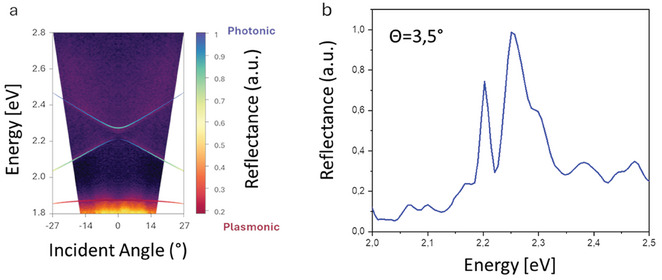
a) Measured reflectance map for incident P_x_ with color‐coded lines representing the eigenvalues calculated by diagonalizing the 3 × 3 Hamiltonian. b) Measured reflectance spectrum illustrating the resonance of the hybrid quasi‐BIC mode extracted at θ   = 3.5°.

Equation (1) gives rise to three hybrid modes: one upper branch (indicated as UB), one middle branch (MB), and one lower branch (LB). Notably, by fitting the modes position with the Hamiltonian eigenvalues, we found *E_LSPR_* ≈ 1.9 eV and we extracted a Rabi splitting of Ω ≈200 meV. In **Figure** **3**a are shown the polariton dispersions where the hybrid modes appear as a function of a colour‐coded plasmonic/photonic fractions, calculated by the Hopfield coefficients for the branches around the plasmon energy. These fractions are overlapped to the corresponding measured reflection map (also shown in Figure S7, Supporting Information without overlapping).

Interestingly, while the UB is affected to a lesser extent by the LSPR position (exhibiting a more photonic character), a stronger coupling involves the MB and LB modes. Ultimately, the balance between photonic and plasmonic content dictates the coupling regime. This interplay governs whether the system operates in the weak coupling regime, where modes retain their individual identities, or in the strong coupling regime, where hybrid modes emerge due to significant interaction between photonic and plasmonic fields. Adjusting this balance through careful design and tuning of the lattice period is found essential for tailoring the optical properties toward our specific application, as detailed in the next section.

Figure 3b shows a representative experimental reflectance spectrum evidencing the quasi‐BIC resonance with a FWHM of 3.5 nm, i.e., an intrinsic Q‐factor of 160 at 3.5°.

### 2.2 Mode Volume (Field Enhancement) Versus Q Factor

**Figure 4:**
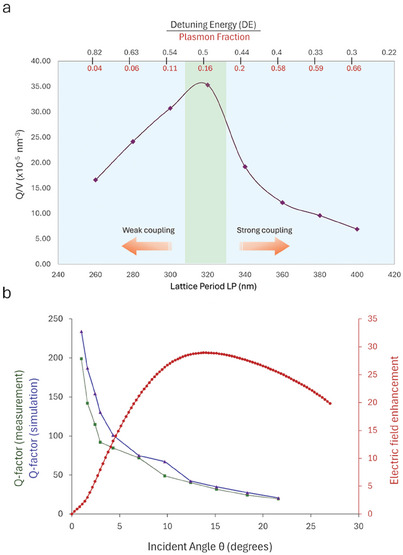
a) Q/V ratio trend calculated for several lattice periods (along the dimers axis) as a function of both the detuning of energy (DE) between the LSPR and photonic BIC modes and the plasmon fraction derived from the Hopfield coefficients computation. The maximum value of Q/V is found at LP = 320 nm, corresponding to a DE = 0.5 eV and a 16% plasmon fraction participating in the hybrid branch. b) Correlation among the simulated and measured Q‐factor values and calculated electric field enhancement in the optimal DE region (320 nm lattice period along the x‐axis) as a function of the incident angle.

For a more in‐depth analysis, we extracted the theoretical Q‐factors and the modal volumes of the hybrid quasi‐BIC as a function of the DE value, varying the lattice period (LP) of the dimers along P_x_ and producing a spectral scan of the photonic branches on the LSP mode, as shown in **Figure** **4**a (the corresponding simulated reflection maps are shown in Figure S3, Supporting Information). A definition of modal volume V_mod_ can be given in terms of the electric energy density $u_{E}=\frac{1}{2}\;\varepsilon_{r}\varepsilon_{0}{|\vec{E}|}^{2}$ in which $\varepsilon_{r}$ is the relative permittivity, as

(2) $$V_{mod}=\frac{\int_{\Xi }u_{E}\;dV}{u_{E}^{max}}$$

where Ξ formally extends to infinity in the direction orthogonal to the array plane and $u_{E}^{max\;}$ is the maximum energy density within the same region of interest.^[^ ^41^ ^]^ Considering the difficulty in calculating the mode volume, we defined an effective modal volume V_eff_ ranging between the bottom and the top surfaces of the NDs, and whose base area covers the entire unit cell of the array.^[^ ^43^, ^44^ ^]^

The reflectance spectrum at the peak was fitted to a Fano shaped resonance (Figure S6, Supporting Information), and the full width at half‐maximum close to the peak center at the given λ was used to determine the Q‐factor, which was calculated as $\frac{\lambda }{\Delta \lambda }$.

According to what is shown in Figure 4a, smaller LP values induce a strong blueshift of the BIC mode, significantly increasing the DE with the LSP mode (weak coupling regime) and exhibiting a more photonic behavior, as a BIC‐like diffractive order. As a result, the Q‐factor values in this region are very high but the large modal volumes do not allow to reach an optimal *Q/V* ratio peaked at LP = 320 nm. On the other hand, large LPs enable the full spectral and spatial overlap between the two bare modes (strong coupling regime). Here, the prevailing plasmonic fraction leads to very small modal volumes but that's not sufficient to compensate for the Q values decrease, which overall results in Q/V dropping.

Therefore, following these results, we defined an intermediate DE region for an optimum balance between large Q‐factor and ultrasmall V coincident with the P_x_ case in our experiment.^[^ ^18^ ^]^ These results could be explained also by Hopfield coefficient values as calculated in Figure S7 (Supporting Information) Indeed, a sufficiently low plasmonic fraction (16%), corresponding to a DE = 0.5 eV, ensures the hybrid BIC onset, with maximized Q/V ratio.

When switching the polarization along the y‐axis, the optical behavior resembles the condition of strong coupling identified in Figure 4a but with the distinction that in this case, as earlier mentioned and shown in Figure 2b,d, the hybrid BIC generation is compromised due to reduced electric field strength of the single ND and the high LSP losses acting as an additional destructive leaky channel.

To better evidence the photonic features of the hybrid modes, in Figure 4b we show the experimental and simulated Q values of the hybrid MB as a function of the incident angle. Approaching the **Γ**‐point at θ  = 0°, the *Q*‐factor exhibits a burst. However, the ability to confine and enhance light at the quasi‐BIC resonance is very crucial for light‐matter interaction. Here, the light confined at this regime, unlike regular guided modes below the light line, can be optimally excited by the free propagating plane waves only at the critical coupling regime. Therefore, the plasmonic dimer with its radiative dipole resonance offers a tool to inject energy into the system and balance at the same time the ohmic nonradiative loss of silver, but only at a certain nonzero momentum owing to its significantly increased radiative quality factor at normal incidence. Indeed, evaluating the local field enhancement |E/E_o_|^2^ averaged over the unit cell as a function of the incident angle, as illustrated in Figure 4b, the field enhancement has a maximum at ≈13°, whereas the Q‐factor shows a maximum value up to 200 at normal incidence.

### 2.3 Hybrid Quasi BIC Resonance Tracking for Biosensing

**Figure 5:**
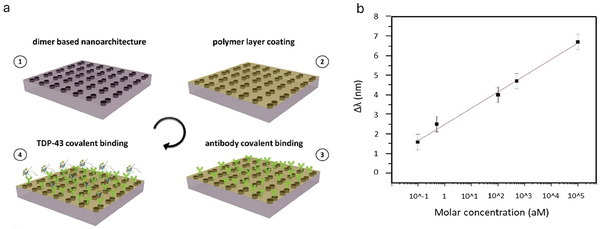
a) Schematic representation of the functionalization process and biomolecule binding: 1‐ dimer‐based nanoarchitecture, 2‐ polymer layer coating, 3‐ antibody covalent binding, 4‐ target analyte TDP‐43 covalent binding to its specific antibody. b) Relationship between the spectral difference between PBS (reference) and TDP‐43 as a function of the molar concentration (LoD) detected by hybrid SP‐BIC resonance tracking.

To test the potential of the proposed platform for biosensing applications, we have employed an engineered functionalized polymer with high yield coverage and specific biorecognition of target molecules of a neurodegenerative related biomarker. A schematic representation of the functionalization process is described in **Figure** **5**a while details can be found in the experimental section. At first, the fabricated array (1) was uniformly coated by an ultrathin (≈10 nm) layer of polydopamine by self‐polymerization. The presence of the ultrathin polymer does not alter how the hybrid BIC field interacts with molecules (2),^[^ ^45^ ^]^ as it does not substantially change the distribution, skin depth, and intensity of the plasmonic field and the resulting refractive index shift, induced by the polymer deposition, does not induce a relevant detuning of the bare modes, preserving the spectral and spatial coupling conditions in the hybrid modes generation Then we exploited the high‐density chemical moieties of the polymer for antibody covalent binding (3). In the end, as a proof‐of‐principle, we tested the sensor's response to different concentrations of TDP‐43, a biomarker related to amyotrophic lateral sclerosis (ALS) and other neuropathies (Figure S9, Supporting Information). To validate the repeatability of the experiment, four measurements were acquired on four different arrays. As shown in Figure 5b, we observed a resonance shift proportional to the analyte concentration ranging from 100 femtomolar to 100 zeptomolar and our sensor proved to be able to detect TDP‐43 down to 100 zm. In Figure S8 (Supporting Information) shows more in detail of the resonance shifts’ identification procedure, while Figure S10 (Supporting Information) shows the sensitivity measured for different glycerol‐water concentrations. To the best of our knowledge, this result goes beyond the interval accessible for this analyte through typical immunoassays,^[^ ^46^, ^47^, ^48^ ^]^ thus representing the first biosensing signature reaching this concentration level.

The sensing performances arise from the desired combination between the fundamental structural configuration and the ultrathin polymer layer. In particular, the dimer‐based nanosystem accommodates the onset of the hybrid plasmonic‐photonic SP‐BIC exhibiting q‐BIC with maximized Q/V ratio and a strong EM field enhancement both amplified by the nanogap effect. On the other hand, the polymer layer positively acts in a threefold manner: it prevents silver's chemical tarnishing,^[^ ^49^ ^]^ it guarantees direct access to the hybrid quasi‐BIC field for the strong interaction with the target molecules and allows for a high‐density immobilization of antibodies. The performance of the dimer‐based nanosystem surpasses those of previous works based on quasi‐BICs as shown in Table S1 (Supporting Information).

Finally, we evaluated the bulk refractometric sensitivity by varying the cladding index using glycerol/water solutions at different relative concentrations, as illustrated in Figure S10 (Supporting Information), which indicated a significant sensitivity of 1100 nm/ RIU. A comparison with the state of the art for relatively similar dimer‐based Systems^[^ ^17^, ^41^, ^50^, ^51^, ^52^, ^53^, ^54^, ^55^, ^56^ ^]^ confirms the large potentiality for the proposed biosensing approach.

## 3 Conclusion

We envisaged a new functional optical platform combining photonic mode and LSP in a non‐trivial 2D nanoarchitecture to build large light‐matter interactions in a hybrid SP‐BIC. The engineered building block sets the optical conditions to enter the coupling regime between LSP and quasi‐guided modes, unlocking hybrid BIC states with high Q‐factor values and small modal volume (boosted EM field enhancement). We reported that the hybrid BIC onset strongly depends on the spectral and spatial overlap of the bare modes but especially on the huge intensity of the plasmonic field. The latter is made possible only by the gap hot spot activation for proper incident polarization (aligned with the axis dimer). In addition, we identified a region of energy detuning between the bare plasmonic and photonic modes that grants the best trade‐off in terms of Q/V by fine‐tuning the photonic‐to‐plasmonic fraction involved in the strong coupling, scanning the coupling strength as a function of the lattice period. Not only the radiation loss is significantly damped by the emergence of the quasi BIC, but this holds true in the periodic array of plasmonic gap hot spots, which leads to small modal volumes beneficial for light‐matter interaction enhancement. The proposed metasurface provides a further pivotal feature: the dimer top surface is exposed to the environment, thus ensuring direct access to the active surfaces which is a crucial requirement for high light‐matter interaction. We have exploited this novel design in a proof‐of‐concept optical biosensing experiment and it proved to be highly effective in the detection of TDP‐43, a biomarker related to amyotrophic lateral sclerosis (ALS) and other neuropathies. Strikingly, we were able to reach a 100 zeptomolar concentration, which constitutes an unprecedented level of bio‐molecular sensing capability. All the above‐mentioned characteristics can be well‐tuned by tailoring the geometry of the nanostructures. Overall the configurational features of our nanoarchitecture provide multifunctional properties thus paving the way to versatile hybrid light matter optical devices for various applications such as lasing and biosensing. Such a study lays the foundation for the implementation of an innovative architecture as an advanced biosensing platform. Unlike other Γ‐BIC based nanosystems, here a multifunctional approach opens the way toward the simultaneous enforcement of multiple independent targets, i.e., maximizing the light‐matter coupling and reaching high performances in biosensing, by means of a specific detuning between the BIC energy valley and the LSPR that creates the conditions for losses suppression.

## 4 Experimental Section

### Materials and Methods

Dopamine hydrochloride ((HO)_2_C_6_H_3_CH_2_CH_2_NH_2_HCl, powder, Sigma–Aldrich, USA), and polyclonal antibody anti‐TDP‐43 (liquid, Proteintech, USA) were used as received. A stock solution of TDP‐43 (5 mm) was prepared in phosphate buffer solution at pH 7.4 and stored at −20° C if not used. Standard stock solution of dopamine hydrochloride (0.1 mg mL^−1^) was prepared in Tris‐HCl buffer solution (10 mm, pH 8.5). Phosphate‐buffered saline (PBS), and tris(hydroxymethyl) aminomethane hydrochloride (Tris‐HCl) were prepared by using reagent‐grade salts purchased from Sigma. All solvents, purchased from Sigma–Aldrich, were of the highest purity available. All aqueous solutions were prepared by using water obtained from a milli‐Q Gradient A‐10 system (Millipore, 18.2 MΩ cm, organic carbon content ≤ 4 µg L^−1^).

### Biomolecule Binding

Specific molecular detection using the fabricated metasurface has been realized, which involved three steps: surface activation, probe immobilization, and target capture. First of all, an ultrathin PDA layer was formed on the metasurface by keeping a solution of dopamine hydrochloride 0.1 mg mL^−1^ in Tris‐HCl buffer at pH 8.5 overnight. In these conditions, the PDA catechol groups, responsible for adhesion on substrates, were oxidized to o‐quinone, which was responsible for cross‐linking.^[^ ^57^ ^]^ The PDA coated metasurface was then rinsed with milli‐Q water and dried under nitrogen flow. The bioconjugation reaction with TDP‐43 was performed by exploiting the ability of PDA to immobilize biomolecules through a reaction with ‐NH_2_ groups, by simple incubation with an antibody solution 5 mm in buffer Tris‐HCl at pH 8.5 for 1h. At alkaline pH, catechol and quinone functional groups present in the polydopamine coating were capable of covalent coupling to nucleophiles, thus allowing subsequent biomolecule immobilization through reaction between amines and polydopamine‐coated substrates.^[^ ^58^ ^]^ Then, the platform was rinsed with water and dried under nitrogen flow. Finally, the sensing performances of the fabricated nanosensor were investigated by incubating them with solutions of the un‐tagged human recombinant TDP‐43 protein at different concentrations ranging from 100 fM to 100 zm in PBS for 40 min. The device was then rinsed in milliQ water and finally dried. This measurement procedure was repeated on four different arrays and performed four times on each of them.

### Sample Fabrication

2D 80 × 80 silver nanodisks dimers with 70 nm diameter, 45 nm height, and 30 nm gap were realized on a dielectric Si_3_N_4_ slab by electron beam lithography, with lattice periods 320 × 300 nm. The 210 nm thick layer of Si_3_N_4_ was evaporated via the PECVD process onto a glass substrate. The sample was first cleaned in acetone and isopropyl alcohol. Then a 200 nm poly(methyl methacrylate) (PMMA) layer was spin‐coated at 3000 rpm and soft‐baked at 180 °C for 3 min. A 5 nm thick chrome layer was thermally evaporated onto the PMMA to prevent charge effects in the electron beam writing procedure. The arrays were written by a Raith 150 system at 26 pA beam current and 30 keV. After electron exposure, the Cr layer was completely removed by ceric ammonium nitrate‐based wet etching for 40 s and rinsed in water. The exposed resist was developed in MIBK:IPA solution in a 1:3 ratio for 3 min and rinsed in 2‐propanol for 1 min. The sample was then treated with Inductively Coupled Plasma Etching (ICP) in order to recreate the design that was drawn on the resist onto the Si_3_N_4_ slab. After thermal evaporation of 40 nm of silver, a lift‐off process was performed in an mr‐Rem 500 remover solution (Microresist Technology) and rinsed in 2‐propanol.

### Numerical Simulations

Numerical simulations were performed by exploiting an FTDT and RCWA based software (Ansys Lumerical). Considering the array system investigated, all parametric studies have been performed by simulating an elemental unit cell with an Ag disk dimer embedded in a Si_3_N_4_ waveguide with the top surface exposed to the environment and employing periodic boundary conditions in the array to get energy−momentum reflection maps E(k_x_,k_y_ = 0) for different simulation designs. The glass substrate was not included in the simulations. For what concerns the optical properties of the plasmonic metal involved, the Johnson and Christy formula for the permittivity dispersion of silver was considered. From the environmental point of view, the disks have been considered exposed to a non‐dispersive dielectric medium with refractive index *n* = 1.4 to simulate the effect of the 10 nm layer of polymer.

### Optical Characterization

The sample was characterized by reflection measurements on a home‐made confocal setup comprising an optical microscope (Zeiss AxioScope A1) coupled to a spectrometer. Light from a tungsten lamp was focused on the sample by an adjustable numerical aperture condenser (NA from less than 0.1 to 0.95). Reflected light was collected with a 10 × 0.95 NA objective lens. Subsequently, the light passes through a three lenses system: the first reconstructs the real space, the second collimates the light beam, and the third lens refocuses the image in the real space. The light was then directed to a Hamamatsu Orca R2 CCD camera and a 150 mm spectrometer. By using all the three lenses combined with an additional pinhole, the image can be selected in space. Removing the intermediate lenses, instead, Fourier space imaging had been obtained. An adjustable pinhole was used to select the array area in the focal plane.

## Conflict of Interest

The authors declare no conflict of interest.

## Supporting information



Supporting Information

## Data Availability

The data that support the findings of this study are available from the corresponding author upon reasonable request.

## References

[1] C. W. Hsu , B. Zhen , A. D. Stone , J. D. Joannopoulos , M. Soljačić , Nat. Rev. Mater. 2016, 1, 16048.

[2] H. Zhong , T. He , Y. Meng , Q. Xiao , Materials 2023, 16, 7112. 38005042 10.3390/ma16227112 PMC10672634

[3] E. De Tommasi , S. Romano , V. Mocella , F. Sgrignuoli , V. Lanzio , S. Cabrini , G. Zito , Adv. Opt. Mater. 2023, 11, 2300475.

[4] A. Kodigala , T. Lepetit , Q. Gu , B. Bahari , Y. Fainman , B. Kanté , Nature 2017, 541, 196. 28079064 10.1038/nature20799

[5] S. Mohamed , J. Wang , H. Rekola , J. Heikkinen , B. Asamoah , L. Shi , T. K. Hakala , Laser Photonics Rev. 2022, 16, 2100574.

[6] J. M. Foley , S. M. Young , J. D. Phillips , Phys. Rev. B 2014, 89, 165111.

[7] G. Yang , S. U. Dev , M. S. Allen , J. W. Allen , H. Harutyunyan , Nano Lett. 2022, 22, 2001. 35175777 10.1021/acs.nanolett.1c04764

[8] M. Luo , Y.i Zhou , X. Zhao , Z. Guo , Y. Li , Q.i Wang , J. Liu , W. Luo , Y. Shi , A.i Q. Liu , X. Wu , ACS Nano 2024, 18, 6477. 38350867 10.1021/acsnano.3c11994

[9] S. I. Azzam , A. V. Kildishev , Adv. Opt. Mater. 2022, 9, 2001469.

[10] K. Koshelev , S. Lepeshov , M. Liu , A. Bogdanov , Y. Kivshar , Phys. Rev. Lett. 2018, 121, 193903. 30468599 10.1103/PhysRevLett.121.193903

[11] C. Schiattarella , S. Romano , L. Sirleto , V. Mocella , I. Rendina , V. Lanzio , F. Riminucci , A. Schwartzberg , S. Cabrini , J. Chen , L. Liang , X. Liu , G. Zito , Nature 2024, 626, 765. 38383627 10.1038/s41586-023-06967-9 PMC10881401

[12] A. Aigner , T. Weber , A. Wester , S. A. Maier , A. Tittl , Nat. Nanotechnol. 2024, 19, 1804. 39187580 10.1038/s41565-024-01767-2 PMC11638065

[13] R. Chikkaraddy , B.d. Nijs , F. Benz , S. J. Barrow , O. A. Scherman , E. Rosta , A. Demetriadou , P. Fox , O. Hess , J. J. Baumberg , Nature 2016, 535, 127. 27296227 10.1038/nature17974 PMC4947385

[14] S. Romano , G. Zito , S. Managò , G. Calafiore , E. Penzo , S. Cabrini , A. C. De Luca , V. Mocella , J. Phys. Chem. C 2018, 122, 19738.

[15] M. H. Chowdhury , J. Pond , S. K. Gray , J. R. Lakowicz , J. Phys. Chem. C 2008, 112, 11236. 10.1021/jp802414k PMC2748429 19777130

[16] Y. Jeong , Y.‐M. Kook , K. Lee , W.‐G. Koh , Biosens. Bioelectron. 2018, 111, 102. 29660581 10.1016/j.bios.2018.04.007

[17] S. Sarkar , T. A. F. König , Adv. Sens. Res. 2024, 3, 2300054.

[18] M. Manoccio , V. Tasco , F. Todisco , A. Passaseo , M. Cuscuna , I. Tarantini , G. Gigli , M. Esposito , Adv. Sci. 2023, 10, 2206930. 10.1002/advs.202206930 PMC9951338 36575146

[19] I. C. Seo , S. Kim , B. H. Woo , I.‐S. Chung , Y. C. Jun , Nanophotonics 2020, 9, 4565.

[20] A. Aigner , A. Tittl , J. Wang , T. Weber , Y. Kivshar , S. A. Maier , H. Ren , Sci. Adv. 2022, 8, eadd4816. 36490330 10.1126/sciadv.add4816 PMC9733921

[21] Z. Wang , Y. Liang , J. Qu , M. K. Chen , M. Cui , Z. Cheng , J. Zhang , J. Yao , S. Chen , D. P. Tsai , C. Yu , Photonic Research 2023, 11, 260.

[22] V. Kabashin , A. G. Kravets , N. Grigorenko , Chem. Soc. Rev. 2023, 52, 6554. 37681251 10.1039/d3cs00155e

[23] M. Luo , Y. Zhou , X. Zhao , Y. Li , Z. Guo , X. Yang , M. Zhang , Y. Wang , X. Wu , Biosensors 2022, 12, 1120. 36551087 10.3390/bios12121120 PMC9775062

[24] Z. Wang , J. Sun , J. Li , L. Wang , Z. Li , X. Zheng , L. Wen , Adv. Sci. 2023, 10, 2206236. 10.1002/advs.202206236 PMC9982570 36594610

[25] Y. Jahani , E. R. Arvelo , F. Yesilkoy , K. Koshelev , C. Cianciaruso , M. De Palma , Y. Kivshar , H. Altug , Nat. Commun. 2021, 12, 3246. 34059690 10.1038/s41467-021-23257-y PMC8167130

[26] G. Zito , G. Sanità , B. Guilcapi Alulema , S. N. Lara Yépez , V. Lanzio , F. Riminucci , S. Cabrini , M. Moccia , C. Avitabile , A. Lamberti , V. Mocella , I. Rendina , S. Romano , Nanophotonics 2021, 10, 4279.

[27] J. Wang , J. Kühne , T. Karamanos , C. Rockstuhl , S. A. Maier , A. Tittl , Adv. Funct. Mater. 2021, 31, 2104652.

[28] A. Ndao , L. Hsu , W. Cai , J. Ha , J. Park , R. Contractor , Y. Lo , B. Kanté , Nanophotonics 2020, 9, 1081.

[29] C. Schiattarella , G. Sanità , B. G. Alulema , V. Lanzio , S. Cabrini , A. Lamberti , I. Rendina , V. Mocella , G. Zito , S. Romano , Biosens. Bioelectron. X 2022, 12, 100262.

[30] Y. F. Xiao , Y. C. Liu , B. B. Li , Y. L. Chen , Y. Li , Q. Gong , Phys. Rev. A 2012, 85, 031805.

[31] S. Joseph , S. Sarkar , S. Khan , J. Joseph , Adv. Opt. Mater. 2021, 9, 2001895.

[32] M. Meudt , C. Bogiadzi , K. Wrobel , P. Görrn , Adv. Opt. Mater. 2020, 8, 2000898.

[33] Q. T. Trinh , S.y K. Nguyen , D. H. Nguyen , G. K. Tran , V. H. Le , H. S. Nguyen , Q. Le‐Van , Opt. Lett. 2022, 47, 1510. 35290351 10.1364/OL.447933

[34] S. I. Pavlov , I. M. Fradkin , S. A. Dyakov , N. A. Feoktistov , A. V. Nashchekin , A. B. Pevtsov , Phys. Rev. B 2023, 108, 205425.

[35] Z. F. Sadrieva , I. S. Sinev , K. L. Koshelev , A. Samusev , I. V. Iorsh , O. Takayama , R. Malureanu , A. A. Bogdanov , A. V. Lavrinenko , ACS Photonics 2017, 4, 723.

[36] S. I. Azzam , V. M. Shalaev , A. Boltasseva , A. V. Kildishev , Phys. Rev. Lett. 2018, 121, 253901. 30608828 10.1103/PhysRevLett.121.253901

[37] Y. Liang , K. Koshelev , F. Zhang , H. Lin , S. Lin , J. Wu , B. Jia , Y. Kivshar , Nano Letters 2020, 20, 6351. 32479094 10.1021/acs.nanolett.0c01752

[38] X. Zhao , L. Xiong , Z. Zhang , G. Li , Opt. Express 2022, 30, 34601. 36242469 10.1364/OE.471356

[39] S. T. Ha , Y. H. Fu , N. K. Emani , Z. Pan , R. M. Bakker , R. Paniagua‐Domínguez , A. I. Kuznetsov , Nat. Nanotechnol. 2018, 13, 1042. 30127475 10.1038/s41565-018-0245-5

[40] H. Sigurðsson , H. C. Nguyen , H. S. Nguyen , Nanophotonics 2024, 13, 3503. 39185487 10.1515/nanoph-2023-0834 PMC11341133

[41] F. Todisco , M. Esposito , S. Panaro , M. De Giorgi , L. Dominici , D. Ballarini , A. I. Fernández‐Domínguez , V. Tasco , M. Cuscunà , A. Passaseo , C. Ciracì , G. Gigli , D. Sanvitto , ACS Nano 2016, 10, 11360. 28024373 10.1021/acsnano.6b06611

[42] P. Vaity , V. G. Achanta , Conference on Lasers and Electro‐Optics JW1A.177 (Optica Publishing Group, San Jose, California, 2021).

[43] C. Sauvan , J. P. Hugonin , I. S. Maksymov , P. Lalanne , Phys. Rev. Lett. 2013, 110, 237401. 25167528 10.1103/PhysRevLett.110.237401

[44] P. T. Kristensen , C. V. Vlack , S. Hughes , Opt. Lett. 2012, 37, 1649. 22627525 10.1364/OL.37.001649

[45] G. Zito , G. Siciliano , A. Seifalinezhad , B. Miranda , V. Lanzio , A. Schwartzberg , G. Gigli , A. Turco , I. Rendina , V. Mocella , E. Primiceri , S. Romano , Adv. Sci. 2024, 11, 2401843. 10.1002/advs.202401843 PMC11538715 39236340

[46] E. Feneberg , E. Gray , O. Ansorge , K. Talbot , M. R. Turner , Mol. Neurobiol. 2018, 55, 7789. 29460270 10.1007/s12035-018-0947-6 PMC6132775

[47] Y. Dai , C. Wang , L. Y. Chiu , K. Abbasi , B. S. Tolbert , G. Sauvé , Y. Yen , C. C. Liu , Biosens. Bioelectron. 2018, 117, 60. 29885581 10.1016/j.bios.2018.05.060 PMC6082407

[48] M. Manoccio , M. Esposito , E. Primiceri , A. Leo , V. Tasco , M. Cuscunà , D. Zuev , Y. Sun , G. Maruccio , A. Romano , A. Quattrini , G. Gigli , A. Passaseo , Nano Lett. 2021, 21, 6179. 34251835 10.1021/acs.nanolett.1c01791

[49] M. Scuderi , M. Esposito , F. Todisco , D. Simeone , I. Tarantini , L. De Marco , M. De Giorgi , G. Nicotra , L. Carbone , D. Sanvitto , A. Passaseo , G. Gigli , M. Cuscunà , J. Phys. Chem. C 2016, 120, 24314.

[50] Y. Zhou , B. Wang , Z. Guo , X. Wu , Nanomaterials 2019, 9, 837. 31159384 10.3390/nano9060837 PMC6631114

[51] S. Sarkar , V. Gupta , M. Kumar , J. Schubert , P. T. Probst , J. Joseph , T. A. F. König , ACS Appl. Mater. Interfaces 2019, 11, 13752. 30874424 10.1021/acsami.8b20535 PMC6463243

[52] A. E. Schlather , N. Large , A. S. Urban , P. Nordlander , N. J. Halas , Nano Lett. 2013, 13, 3281. 23746061 10.1021/nl4014887

[53] H. Wang , Scientific Rep. 2018, 8, 9589. 10.1038/s41598-018-28011-x PMC6018101 29941992

[54] J. A. Riley , M. Horák , V. Křápek , N. Healy , V. Pacheco‐Peña , Nanophotonics 2023, 12, 3895. 39635192 10.1515/nanoph-2023-0317 PMC11501113

[55] M. Gittinger , K. Höflich , V. Smirnov , H. Kollmann , C. Lienau , M. Silies , Nanophotonics 2020, 9, 401.

[56] M. M. Mana , B. D. Dana , A. N. Koya , B. Ji , J. Lin , Plasmonics 2024.

[57] G. Siciliano , A. Turco , A. G. Monteduro , E. Fanizza , A. Quarta , R. Comparelli , E. Primiceri , M. L. Curri , N. Depalo , G. Maruccio , Materials 2023, 16, 1697. 36837327 10.3390/ma16041697 PMC9967601

[58] H. Lee , J. Rho , P. B. Messersmith , Adv. Mater. 2009, 21, 431. 19802352 10.1002/adma.200801222 PMC2755254

