# Supplementary material for: Hybrid Plasmonic Symmetry‐Protected Bound state in the Continuum Entering the Zeptomolar Biodetection Range
Source: Small. 2025 Jan 26;21(10):2411827. doi: 10.1002/smll.202411827 (PMC11899489; doi:10.1002/smll.202411827)
Supplement: Supplementary file 1 — Supporting Information [file SMLL-21-2411827-s001.docx]

Supporting Information for

Hybrid plasmonic symmetry-protected bound state in the continuum entering the zeptomolar biodetection range

Elena Clabassi^∥1,2^, Gianluca Balestra^∥1,2^, Giulia Siciliano^1^, Laura Polimeno^1^, Iolena Tarantini^2^, Elisabetta Primiceri^1^, David Maria Tobaldi^1^, Massimo Cuscunà^1^, Fabio Quaranta^3^, Adriana Passaseo^1^, Alberto Rainer^1,5^, Silvia Romano^4^, Gianluigi Zito^4*^, Giuseppe Gigli^1,2^, Vittorianna Tasco^1ǂ^ and Marco Esposito^1,2^*

1. CNR NANOTEC Institute of Nanotechnology, Via Monteroni, Lecce 73100, Italy

2. Department of experimental medicine, University of Salento, Lecce 73100, Italy

3. CNR IMM Institute for Microelectronics and Microsystems, Via Monteroni, Lecce 73100, Italy

4. CNR ISASI Institute of Applied Sciences and Intelligent Systems, Naples 80078, Italy

5. Department of Engineering, University Campus Bio-Medico di Roma, via Álvaro del Portillo 21, 00128 Rome, Italy

*E-mail: [marco.esposito@nanotec.cnr.it](mailto:marco.esposito@nanotec.cnr.it); gianluigi.zito@na.isasi.cnr.it;

∥ E.C. and G.B. contributed equally to this work.

^ǂ^Vittorianna Tasco is currently seconded at the European Research Council Executive Agency of the European Commission. Her views expressed in this paper are purely those of the writer, may not in any circumstance be regarded as stating an official position of the European Commission.

**S1. Photonic BIC in a dielectric slab**


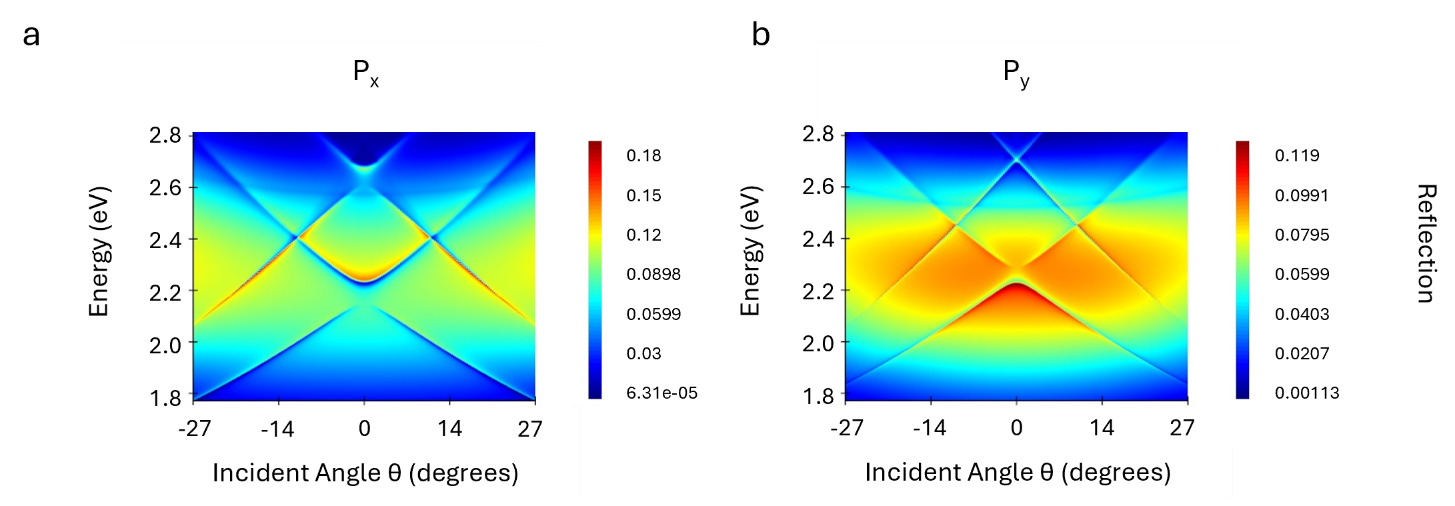


**BIC**

In **Figure S1** we display the the comparison between P_x_ and P_y_ angular dispersion describing the emergence of a purely photonic BIC state from a dielectric Si_3_N_4_ waveguide with a 2D subwavelength grating based on dimers nanoholes. In this scenario, we are able to identify the guided modes because they get diffracted into the radiative continuum thanks to their coupling with Bragg scattering. Along P_x_ (Fig.S1a) the engineered tuning of the nanoholes grating leads to Bloch resonances exhibiting a gap opening and the formation of symmetry-protected BIC at the Γ point of the momentum space. The BIC branch has a maximum in intensity values and appear as dark at normal incidence whereas the “lossy” branch reaches a minimum and is bright. Conversely, along P_y_ (Fig.S1b) we observe a symmetry reversal and the emergence of the BIC mode in the lower branch of the anti-crossed GMs. We note how the gap formed between the hybrid modes is wider in the P_x_ configuration in which also the intensity of the electrical field is higher due to the effect of confinement enhanced by the gap of the dimer.

**S2. Plasmonic grating without waveguide**


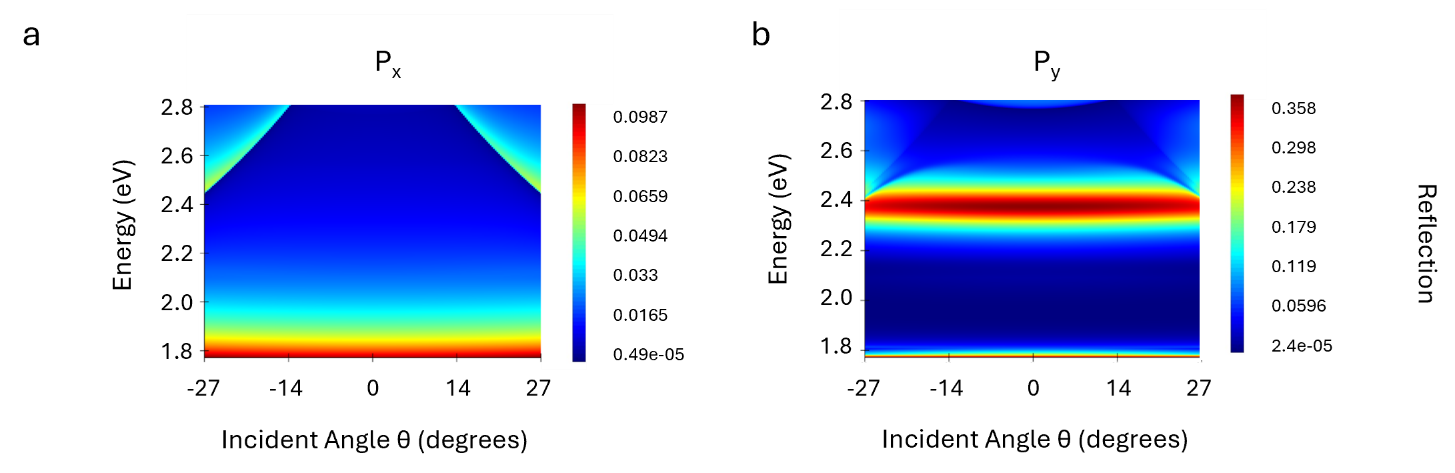


In **Figure S2** are shown the simulations of the angle-resolved reflectance for Ag NDs calculated in a 1.4 refractive index environment (polymer solution) for both the polarizations discussed in the main text. The spectra display the characteristic dispersion branches of the couple of diffractive orders arising from light refraction with the geometry. Additionally, the reflection maps show a single broad LSP resonance: for the dimers configuration it is found at E= 1.7 eV (Fig.S2a), whereas the one related to the single ND appears at E = 2.3 eV (Fig.S2b).

**S3. LP Scanning and Nanogap size variation**


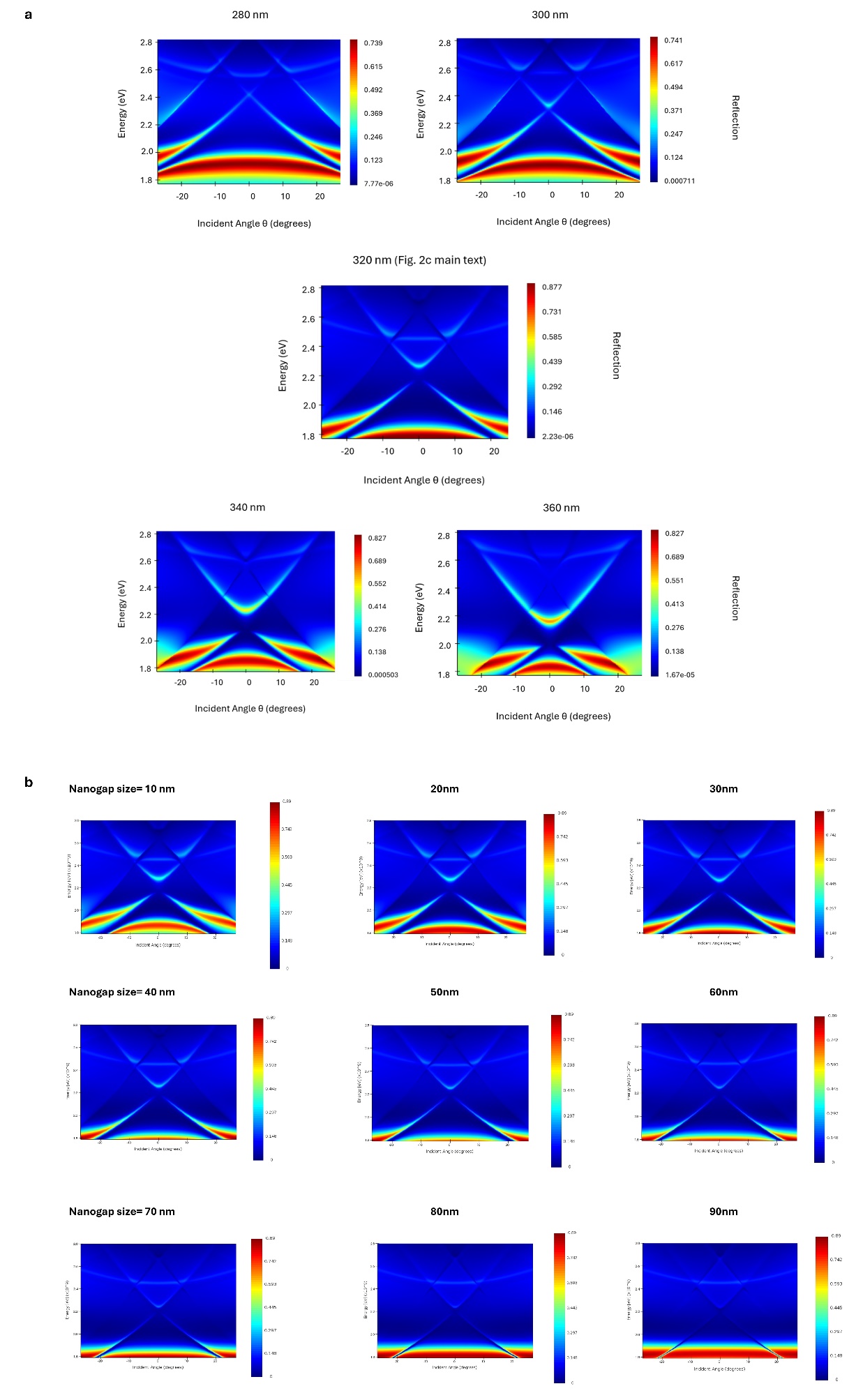


**Figure S3** a) Simulated reflection maps related to the LP scanning along the dimer axis producing a spectral scan of the BIC-like photonic branches on the LSP mode. b) Nanogap size variation from 10nm to 90nm.

For very small LP (280nm) the bare photonic BIC mode is blushifted with respect the LSPR mode, increasing the detuning energy between them and forbidding the formation of hybrid SP-BIC at the gamma point. The LP value increment induces a red shift of the bare photonic mode towards the plasmon energy allowing to the system to enter in the weak coupling regime and the onset of the hybrid BIC with a low plasmonic fraction. As shown in the main text, in this coupling region the optical properties of the nanosystem result optimized for sensing applications. Moving towards higher LP values, the increased overlap between the two bare modes induces a remarkable increment of the plasmonic fraction in the hybrid BIC mode accessing in the strong coupling regime. Here, the optical conditions are suitable for coupling with excitons indicating that this system can be considered as a good candidate for efficient light-matter interactions and future cQED applications.

On the other hand, in the supporting information (**section S3**), we have added simulations that show the impact of the nanogap size variation (from 10nm to 90nm) on the hybrid BIC optical properties. For small nanogap size, the LSP mode is red shifted with respect the photonic BIC allowing to achieve optimal conditions in terms of spatial and spectral overlap, as well as above all high plasmonic field enhancement, in order to trigger the Hybrid BIC. Increasing the nanogap size (90nm), the coupling between the LSP and the photonic BIC modes strongly reduces, forbidding the hybrid BIC trigger. This case resembles to the Py polarization one (in the main text) because the LSP refers to the single isolated ND with reduced electric field strength of the single ND and the high LSP losses acting as an additional destructive leaky channel.

In addition, we note that for large LPs and small nanogap values, the optical bandgap between the hybrid BIC and the lossy modes widens and it is important for strong coupling applications with excitons. Indeed, optimized large optical bandgap would allow to coupling exciton with only hybrid BIC state and decoupling it from the lossy mode increasing the light-matter interaction strength and providing a state with reduced leakages.

**S4. Plasmonic grating on top of the waveguide**

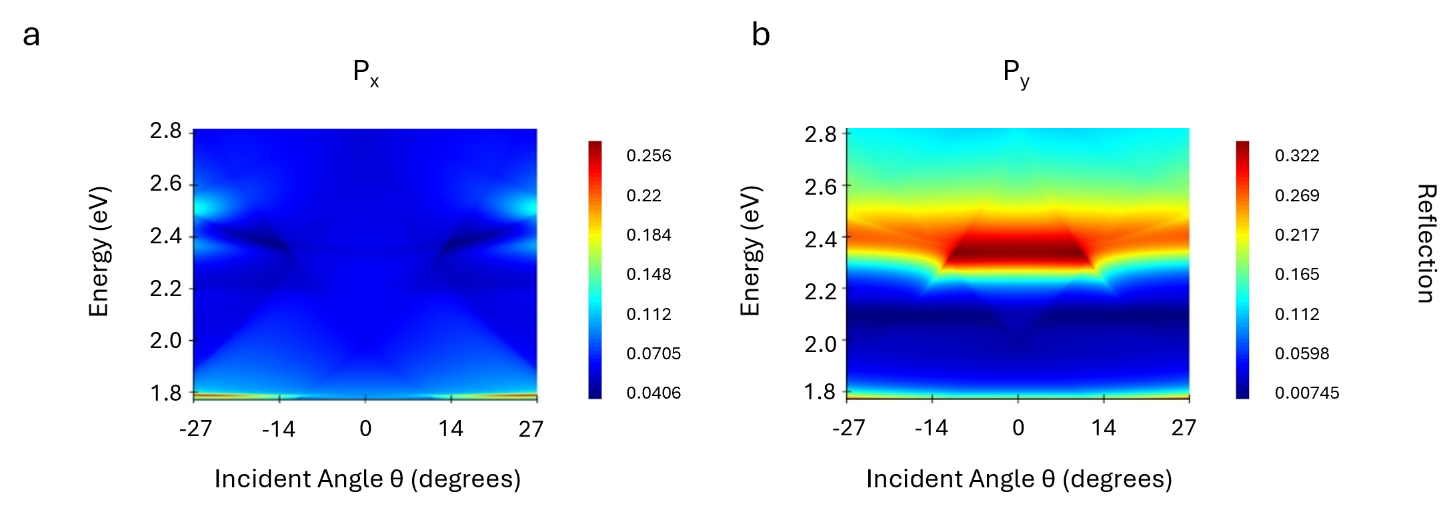


**Figures S4** displays the angle-resolved reflection maps for a Si_3_N_4_ waveguide with a 2D array of silver dimers placed on top and simulated in a 1.4 refractive index environment. Along the dimers polarization axis one can see the dispersions related to diffractive modes of the first and second order together with the LSP resonance related to the dimer at 1.7 eV. Switching to P_y_, one can still find the diffractive modes as mentioned for P_x_, but the LSP resonance is blue-shifted since corresponding to the single ND component. In neither case the hybrid BIC is generated.

**S5. Further numerical simulations to underscore the quasi BIC origin.**

**
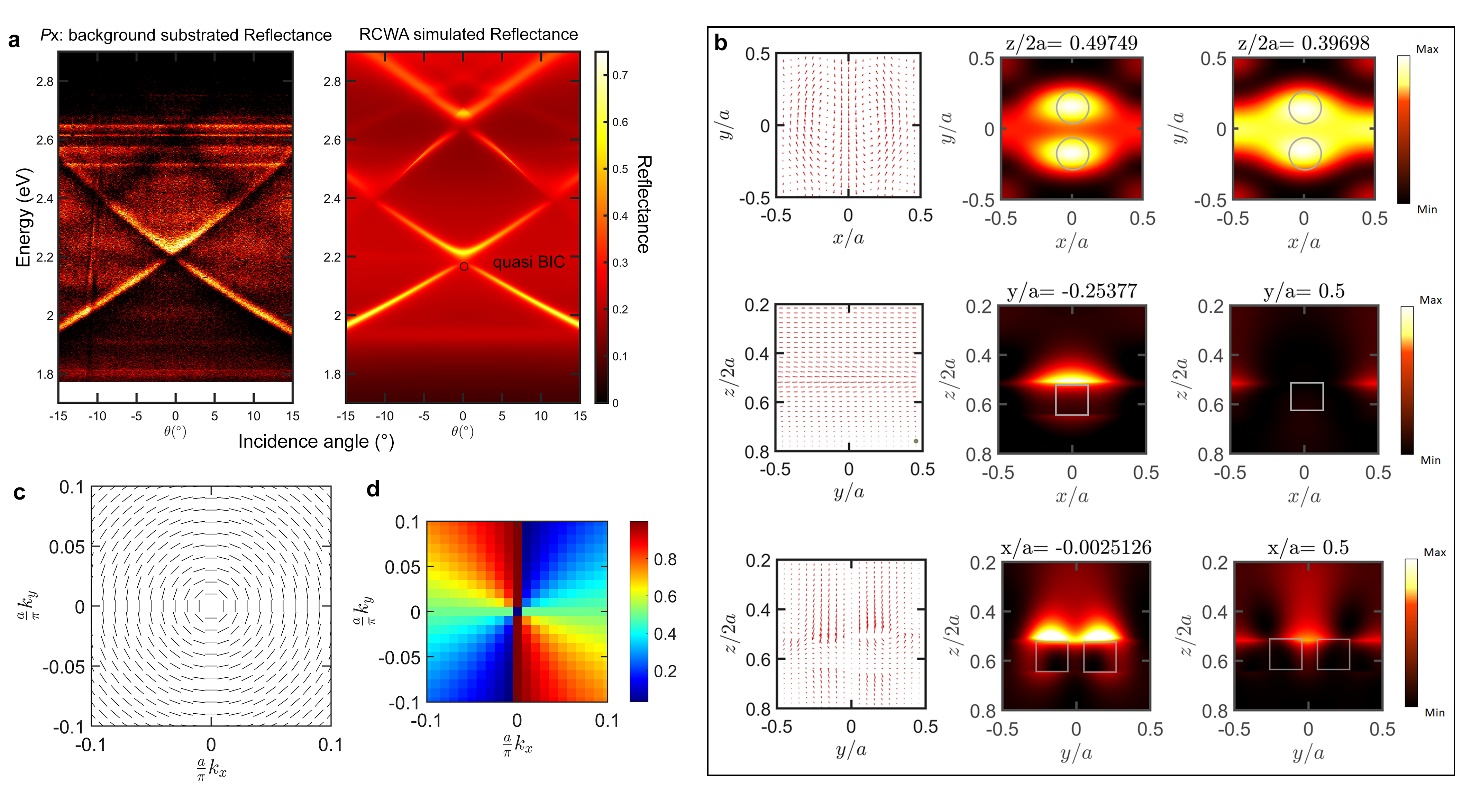
**

**Figures S5** displays in **a** the angle-resolved background-subtracted reflectance map compared with the simulated reflectance calculated with RCWA, showing excellent agreement. **b**, Near field ***E***-vector map (left) and intensity |**E**|^2^ distributions along the three plane cross sections and at the two cut planes indicated on top (middle and right), with coordinates normalized with respect to lattice spacing *a*. The winding of the vector field in closed loops with vortex and anti-vortex pairs, alongside evanescent character in the far field is a signature of quasi BIC. **c**, Eigenpolarization map radiated form the mode shown in **b,** indicating the existence of V-point with topological charge 1 in the azimuthal ellipse angle orientation, consistently with the phase map of the radiated field showing orbital angular momentum equals to 2 (colormap is in units of 2π) in **d**.

**S6. Further numerical simulations to underscore the quasi BIC origin.**


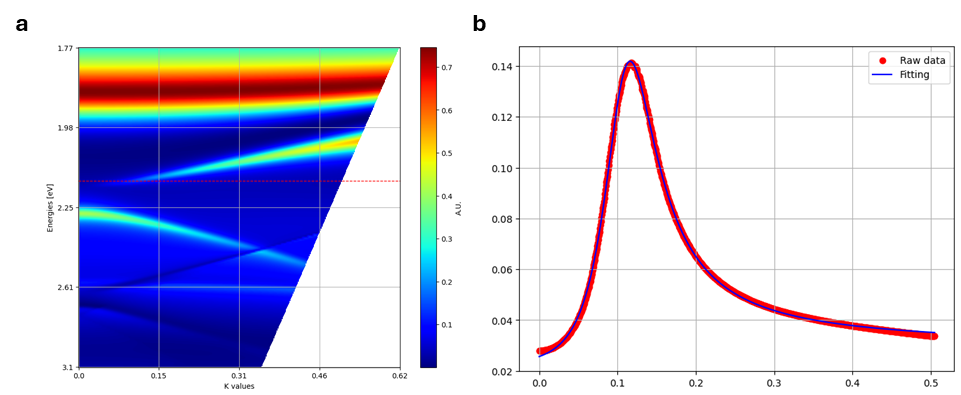


**Figures S6** **a.** The calculated angle-resolved reflectance map. **b.** Reflection spectrum fitted with Fano equation extracted at a fixed energy as indicated by the red line in the **a**.

**S7. Perturbation method**


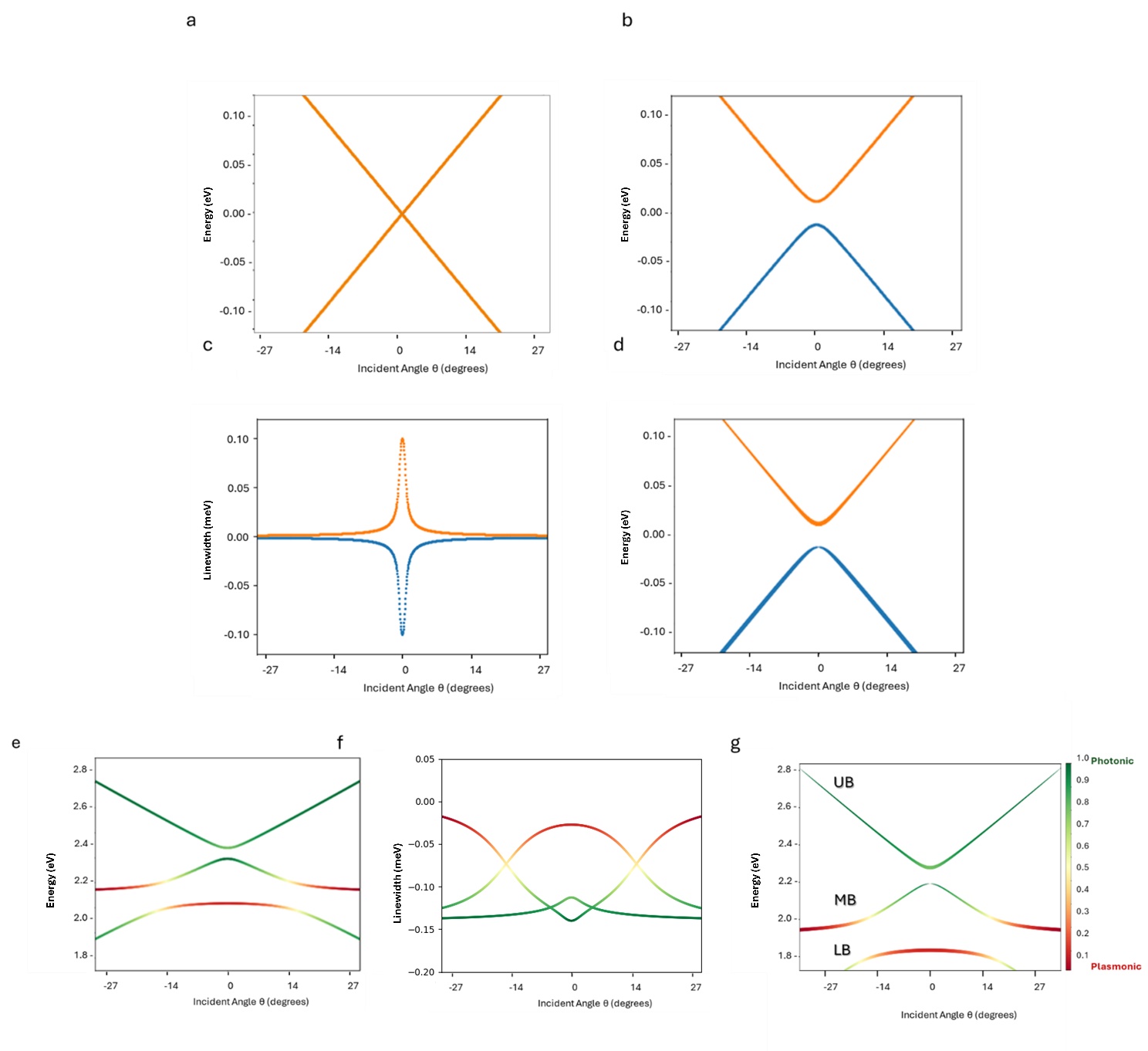


In the main text we have described an analytical model for the coupling regime between the photonic BIC and the LSP resonance based on the calculation of the Hamiltonian for three-coupled oscillators. In **Figure S7** we aim to illustrate the steps that generate the BIC mode from the guided modes, eventually leading to Fig.3a (main text) where the hybridization with the plasmonic component is shown. First, we investigated the behaviour of the fundamental guided modes in the slab. The results are shown in Fig.S5a: presenting only band folding effects, the linear dispersions of the two uncoupled GMs cross one another. Then, we introduced the modifications due to a periodic grating: this induces a diffractive coupling between the modes and consequently the opening of a gap between the resulting Bloch resonances and the formation of SP-BIC at the Γ point of the momentum space. We analyzed this scenario through a two-coupled oscillators system as reported in Eq. 1 below. Here, the term $\gamma$ indicates a matrix whose components take into account the radiative losses in the coupling. In Fig.S5b-c are shown the real and imaginary part, respectively, of the related dispersions. Also, in Fig.S5d is reported the overall dispersions plot to better highlight the formation of the BIC state.

e

f

$H=\left( \begin{matrix} E_{ph}^{+} & g \\ g & E_{ph}^{-} \end{matrix} \right)+i\gamma$ Eq. 1

Now, taking into consideration ^1^, the second step of perturbation consists in the coupling between Bloch resonances and the LSP resonance to form an hybrid plasmonic-photonic SP-BIC. As discussed in the main text (Fig.3a), we approximated this system with a three-coupled oscillators model in which the LSP resonance couples to both the photonic modes relating to the dielectric grating. In Fig. S5e-g are shown the real, imaginary and total components of the dispersions characteristic of the three polariton branches ($\Omega=260 meV,\gamma=0.14 meV)$ .

**S8. Q-bic shift tracking**


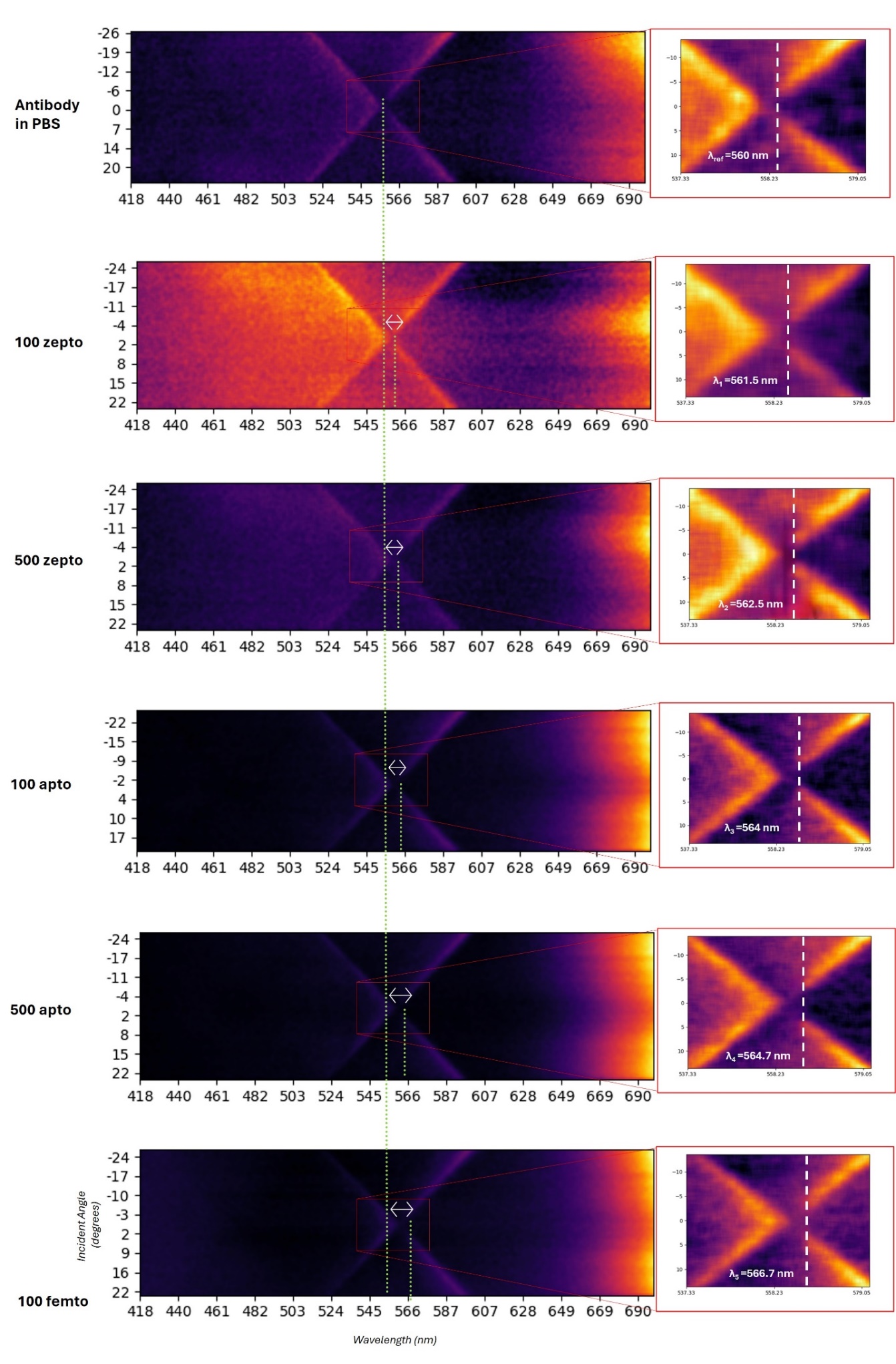


The measured dispersion maps for the antibody and the range of TDP-43 concentrations are shown in **Figure S8**. We considered the quasi-BIC states’ resonance in order to determine the resonance shift attributed to the analyte detection. It’s possible to widely visualize on the maps that these states have shifted in relation to the antibody's one, which is used as a reference. The quasi-BICs can be examined in greater detail from the inlets, and each measurement's associated wavelength is provided. As expected, the highest concentration exhibits the largest resonance shift, but remarkably, a 1.5 nm shift was also present at the 100 zeptomolar concentration, suggesting an unparalleled detection limit for these kinds of biosensing nanostructures.

**S9. Transactive response (TAR) DNA-binding protein 43 (TDP-43)**

A variety of disorders known as neurodegenerative diseases (NDDs) are distinguished by continuing loss and selective malfunctioning of neurons, glial cells, and neural networks in the brain and spinal cord. One of the most common is amyotrophic lateral sclerosis (ALS), a uniformly lethal progressive degenerative disorder of motor neurons that also overlaps with frontotemporal lobar degeneration (FTLD) clinically, morphologically, and genetically^2^. Currently ALS is clinically diagnosed after a typical diagnostic delay of almost a year from the onset of symptoms, by which time the disease is well-established and the window for potential therapy may have closed. In order to address the growing incidence of this disease, it is imperative to create selective and precise biomarkers for early diagnosis and treatment development. In this regard, Transactive response (TAR) DNA-binding protein 43 (TDP-43) has been acknowledged as a potentially useful biomarker. In fact, TDP-43 builds up in nerve cells in most cases of Tau-negative frontotemporal lobar degeneration (FTLD) and almost all cases of ALS ^3,4^ Familial and sporadic ALS and FTLD cases have been linked to the development of TDP-43 mutations. Using mass spectrometry ^5^, Western Blot, and ELISA test ^6,7^, it was discovered that patients with ALS and FTLD had elevated levels of TDP-43 protein in their cerebrospinal fluid (CSF) and plasma. TDP-43 was recently found in CSF at a concentration of less than 0.49 ng/mL via an ELISA test ^8^ and a detection limit of 0.5 ng/mL ^9^ has been reached in serum utilizing an electrochemical sensor. However, most of these methods suffer from drawbacks such as high cost and variability ^10^, complex apparatus requirements, higher limit of detection (LOD), poor reproducibility, and dependence on operator. Furthermore, TDP-43 levels are often below the detection limit of standard immunoassays in a significant portion of both patients and healthy persons ^6,7^. Thus, for a more precise validation of this biomarker and, as a result, for the early diagnosis of ALS and FTLD, more sensitive tests are needed to quantify lower TDP-43 concentrations present in complex fluids.

**Table S1. Sensing performance comparison for quasi-BIC based nanosensors**

| Structures | Sensitivity nm/RIU | FOM | Wavelength | Ref. | Analyte | LOD |
| --- | --- | --- | --- | --- | --- | --- |
| Si3N4 slab (photonic BIC) | NA | NA | visible | A | TGF-β | 10fM |
| plasmonic nanofin (plasmonic BIC) | 6400 | 70 | 5-10um | B | NA | NA |
| Si crescent  (photonic BIC) | 326 | 51.7 | NIR | C | Biotin-streptavidin | 0.167 nM |
| Au/Si3N4  (BIC in Hybrid metasurface) | 492.7 | 266.3 | NIR | D | Biotin-streptavidin | 2.4 nM |
| Au/SiO2 nanoparticle (plasmonic BIC) | 486 | 40.5 | NIR | E | endotoxin | 0.01 EU mL-1 |
| Si diatomic (photonic BIC) | 305 | 68 | Vis/NIR | F | EV | 133 fM |
| Au nanodisk (Plamonic BIC) | 657 | 109 | Vis/NIR | G | NA | NA |
| Si block (Photonic BIC) | 301 | 239 | NIR/IR | H | NA | NA |
| Ag dimer  (Hybrid BIC) | **1100** | **315** | **Visible** | **This work** | **TDP43** | **100zM** |

A. Zito, G.; et al. Molecularly Imprinted Polymer Sensor Empowered by Bound States in the Continuum for Selective Trace‐Detection of TGF‐beta. ADV. Sci. 2024, DOI: 10.1002/advs.202401843

B. Aigner, A.; et al. Plasmonic bound states in the continuum to tailor light-matter coupling. Sci. Adv. 2022, 8, eadd4816.

C. Wang, J.; et al. All-Dielectric Crescent Metasurface Sensor Driven by Bound States in the Continuum. Adv. Funct. Mater. 2021, 31, 2104652.

D. Luo, M.; et al. High-Sensitivity Optical Sensors Empowered by Quasi-Bound States in the Continuum in a Hybrid Metal−Dielectric Metasurface. ACS NANO. 2024, 18, 6477−6486.

E. Wang, Z. C.; Sun, J. C.; Li, J. Y.; Wang, L.; Li, Z. S.; Zheng, X. R.; Wen, L. Y. Customizing 2.5D Out-of-Plane Architectures for Robust Plasmonic Bound-States-in-the-Continuum Metasurfaces. Adv. Sci. 2023, 10, 2206236.

F. Jahani, Y.; Arvelo, E. R.; Yesilkoy, F.; Koshelev, K.; Cianciaruso, C.; De Palma, M.; Kivshar, Y.; Altug, H. Imaging-based spectrometer-less optofluidic biosensors based on dielectric metasurfaces for detecting extracellular vesicles. Nat. Commun. 2021, 12, 3246.

G. Zhou, Y.; Guo, Z. H.; Zhao, X. Y.; Wang, F. L.; Yu, Z. Y.; Chen, Y. Z.; Liu, Z. R.; Zhang, S. Y.; Sun, S. L.; Wu, X. Dual-Quasi Bound States in the Continuum Enabled Plasmonic Metasurfaces. Adv. Opt. Mater. 2022, 10, 2200965.

H. Watanabe, K.; Iwanaga, M. Nanogap enhancement of the refractometric sensitivity at quasi-bound states in the continuum in all-dielectric metasurfaces. Nanophotonics 2023, 12, 99−109.

**S10. Sensitivity**


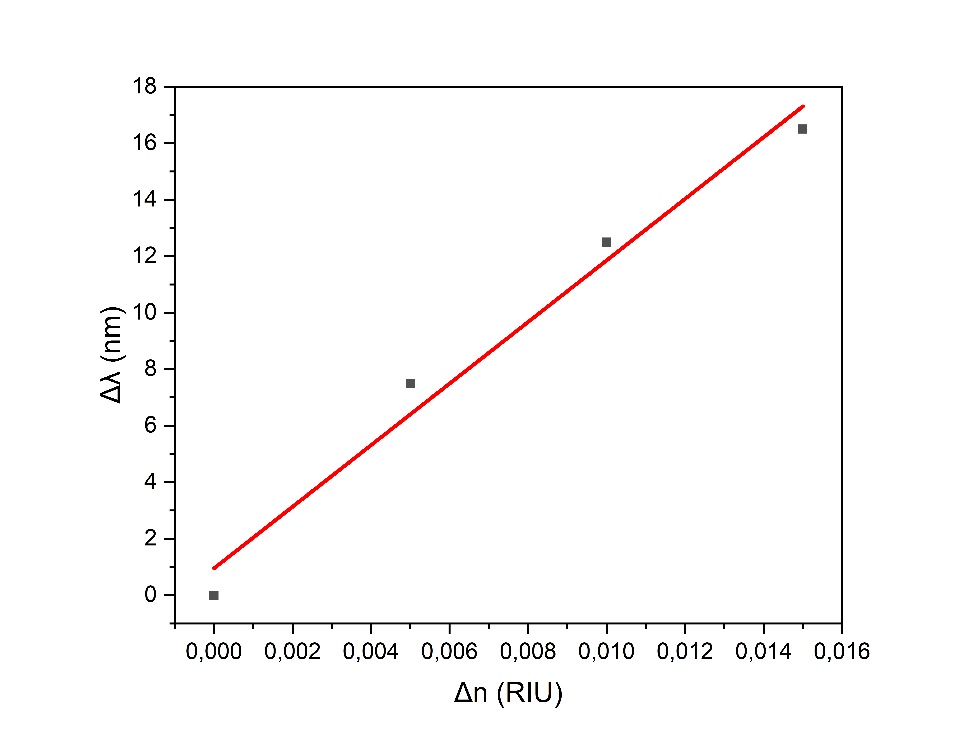


**Figure S10** shows the RI sensitivity curve for different glycerol-water concentrations; we note that the device reaches a sensitivity of 1100nm/RIU.

**References**

1. Lu, L. *et al.* Engineering light-matter strong coupling regime in perovskite-based plasmonic metasurface: quasi-bound state in the continuum and exceptional points. *Photonics Research* **8**, (2020).

2. Parobkova, E. & Matej, R. Amyotrophic Lateral Sclerosis and Frontotemporal Lobar Degenerations: Similarities in Genetic Background. *Diagnostics (Basel)* **11**, 509 (2021).

3. French, R. L. *et al.* Detection of TAR DNA-binding protein 43 (TDP-43) oligomers as initial intermediate species during aggregate formation. *J Biol Chem* **294**, 6696–6709 (2019).

4. Hans, F., Eckert, M., von Zweydorf, F., Gloeckner, C. J. & Kahle, P. J. Identification and characterization of ubiquitinylation sites in TAR DNA-binding protein of 43 kDa (TDP-43). *J Biol Chem* **293**, 16083–16099 (2018).

5. Kametani, F. *et al.* Mass spectrometric analysis of accumulated TDP-43 in amyotrophic lateral sclerosis brains. *Sci Rep* **6**, 23281 (2016).

6. Foulds, P. *et al.* TDP-43 protein in plasma may index TDP-43 brain pathology in Alzheimer’s disease and frontotemporal lobar degeneration. *Acta Neuropathol* **116**, 141–146 (2008).

7. Verstraete, E. *et al.* TDP-43 plasma levels are higher in amyotrophic lateral sclerosis. *Amyotroph Lateral Scler* **13**, 446–451 (2012).

8. Kasai, T. *et al.* Increased TDP-43 protein in cerebrospinal fluid of patients with amyotrophic lateral sclerosis. *Acta Neuropathol* **117**, 55–62 (2009).

9. Dai, Y. *et al.* Application of bioconjugation chemistry on biosensor fabrication for detection of TAR-DNA binding protein 43. *Biosensors and Bioelectronics* **117**, 60–67 (2018).

10. Steinacker, P., Barschke, P. & Otto, M. Biomarkers for diseases with TDP-43 pathology. *Mol Cell Neurosci* **97**, 43–59 (2019).
